# An Important Cause of Blindness in Children: Open Globe Injuries

**DOI:** 10.1155/2016/7173515

**Published:** 2016-05-10

**Authors:** Meral Yildiz, Sertaç Argun Kıvanç, Berna Akova-Budak, Ahmet Tuncer Ozmen, Sadık Gorkem Çevik

**Affiliations:** ^1^Department of Ophthalmology, Uludag University, School of Medicine, 16059 Bursa, Turkey; ^2^Department of Ophthalmology, Şevket Yılmaz Training and Research Hospital, 16310 Bursa, Turkey

## Abstract

*Objective*. Our aim was to present and evaluate the predictive factors of visual impairment and blindness according to WHO criteria in pediatric open globe injuries.* Methods*. The medical records of 94 patients younger than 18 years who underwent primary repair surgery were reviewed retrospectively. The initial and final visual acuity, anterior and posterior segment findings, and zone of injury were noted. The patients were classified as blindness in one eye or visual impairment in one eye.* Results*. Of 412 patients who presented with open globe injury, 94 (23%) were under 18 years old. Fifty-four (16 females, 38 males) children were included. The mean age of the children was 7.1 ± 4.1 years. According to WHO criteria, 19 of 54 patients (35%) had unilateral blindness and 8 had unilateral visual impairment (15%). There was no significant relationship between final visual acuity and gender and injured eye. In visually impaired and blind patients, presence of preoperative hyphema, retinal detachment, and zone 2 and zone 3 injuries was significantly higher.* Conclusion*. Presence of hyphema and zone 2 and zone 3 injuries and retinal detachment may end up with visual impairment and/or blindness in children.

## 1. Introduction

Globally, an estimated 70 million blind person-years are caused by childhood blindness. Approximately 500.000 children become blind every year [1]. In least developing countries congenital and developmental cataract, retinal pathology, and congenital anomalies are the main causes of nontraumatic blindness [2]. Ocular trauma is an important cause of eye morbidity and leading cause of noncongenital monoocular blindness among children [3–5]. Worldwide, eighteen million people have uniocular blindness from traumatic injury and every year a quarter of a million of children present with serious ocular trauma [6]. Two percent to 14% of the pediatric ocular trauma patients ended in visual impairment or blindness [7–9]. In this study, our aim was to present and evaluate the predictive factors of visual impairment and blindness according to WHO criteria in pediatric open globe injuries.

## 2. Methods

Of 412 patients who underwent primary repair surgery because of open globe injury at Department of Ophthalmology, Uludag University, between January 2010 and December 2014, the medical records of 94 patients who were younger than 18 years were reviewed retrospectively. The Uludag University Hospital administration approved the study. The patients younger than 18 years old with at least 6 months of follow-up were included. The exclusion criteria were closed globe injury and major head trauma which might have injured chiasmal and retrochiasmal optic pathways. The initial and final examinations of the patients were evaluated. The initial visual acuity, final visual acuity, anterior and posterior segment findings, zone of injury, computed tomography, and ultrasound findings were noted.

Zone of injury was classified according to Ocular Trauma Classification Group: zone 1 as wound involvement limited to cornea, zone 2 full thickness wound involving the sclera and within 5 mm from the corneoscleral limbus, and zone 3 as wound involvement posterior to the anterior 5 mm of the sclera [10].

The patients were classified according to WHO criteria as blindness in one eye or visual impairment in one eye. Blindness was defined as presenting distance visual acuity <3/60, VI as 3/60 to 6/18 with available correction according to WHO Vision 2020 Action Plan.

For statistical analysis, SPSS 22 statistical program was used. Pearson Chi-square and Fisher's exact test were performed to compare qualitative data. Pearson correlation analysis was used to assess the relation between the parameters. The statistical significance was set at *P* < 0.01 or *P* < 0.05.

## 3. Results

Of 412 patients who presented to Department of Ophthalmology with open globe injury between January 2010 and December 2014, 94 (23%) were under 18 years old. Fifty-four (16 females, 38 males) children with at least 6 months of follow-up were included in the study. The mean age of the children was 7.1 ± 4.1 years (range: 2–16 years) and the mean follow-up was 16.6 ± 10.4 months (range: 6–46 months). We divided the children into 3 groups according to age as 0–4 (19 children), 5–8 (20 children), and ≥9 years (15 children).

Thirty-seven children (68.5%) referred to the department within 24 hours, 15 (27.8%) within 24–48 hours, and 2 (3.7%) of them after 48 hours following the trauma. There was no relation between timing of surgery and visual outcome (*P* = 0.559).

The visual acuity (VA) of 23 children (42.6%), all under 8 years old, could not be measured at presentation. Two children (3.7%) had VA of no light perception and 12 (22.2%) had VA of light perception. The mean VA of 17 children (31.5%) whose Snellen VA could be measured was 0.2 ± 0.3 (Table 1).

Four children (7.4%) had intraocular foreign body at presentation. All the injuries were of penetrating type; the cause of the injury was unknown in 7 children. Fifty-seven percent of them were ≥9 years old. The cause of injuries is given in Table 2. The injuries related with knife occurred under 9 years old. The injuries related with pencil occurred above 4 years old. Zone 1 injury was in 35 (64.8%), zone 2 in 6 (11.1%), and zone 3 in 13 (24.1%) children, respectively. At presentation, hyphema was noted in 12 (22.2%), iris injury in 15 (27.8%), and retinal detachment (3.7%) in 2 children. Lens injury was noted in 28 (51.9%) children. At surgery, it was noted that of 28 patients 13 had capsular rupture. Following lens aspiration, retinal detachment was observed in 6 patients with capsular rupture during surgery.

All the patients underwent primary repair and during their follow-up underwent further surgeries as required. The primary repair consisted of primary suturing of scleral laceration with 8-0 vicryl stitches or corneal perforation with 10-0 nylon stitches. In 13 cases with capsular rupture, lens aspiration was performed at the time of primary repair without intraocular lens (IOL) implantation. Ultrasonography was performed in all patients postoperatively. Except the 8 patients diagnosed with retinal detachment before and at the time of surgery, there was no retinal detachment. Ten of 13 patients with zone 3 injury and 3 of 6 patients with zone 2 injury had vitreous hemorrhage.

The mean number of surgeries including primary repair was 1.6 ± 0.8. Eight patients who had undergone primary repair and lens aspiration had undergone anterior vitrectomy and secondary IOL implantation. Three patients developed endophthalmitis after primary repair. Two of them had intraocular foreign body. All 3 patients had undergone pars plana vitrectomy.

The final visual acuities of 8 patients (14.8%) were light perception and 4 patients were no light perception (7.4%). The mean final visual acuity of the others was 0.5 ± 0.4 (Table 1). According to WHO criteria, 19 of 54 patients (35%) had unilateral blindness and 8 had unilateral visual impairment (15%). There was no significant relationship between final visual acuity and gender and injured eye. In visually impaired and blind patients, presence of hyphema and retinal detachment was significantly higher. The relationship between decreased vision and initial examination findings was shown in Table 3. There was no statistically significant difference between 3 age groups in terms of visual impairment/blindness rates (*P* = 0.884). Seven of 19 patients with blindness had retinal detachment at presentation. At final examination, 6 had corneal scar with 2 of these also having aphakia, and 2 patients had macular scar. Two patients had phthisis bulbi. In one patient, retinal detachment developed due to PVR formation. One patient had traumatic cataract but his parents did not give consent for the secondary surgery.

Five patients with visual impairment had corneal trauma and aphakia and were planned for secondary IOL implantation and corneal transplantation. Two had retinal scar due to foreign body. One had retinal detachment at presentation.

No significant difference was noted between children with regard to visual impairment or blindness when they are grouped as preschool and school-aged children (*P* = 0.783). There was also no significant relationship between the object that caused the injury and visual outcome.

Eight of 14 patients with initial VA of light perception or below had the same VA at final visit. Three of 23 patients whose initial VA could not have been assessed had final VA of light perception or below (Table 1).

All the patients had occlusion in their fellow eye immediately after the primary repair. At 3rd week postoperatively, their refractive errors were corrected with spectacles. During follow-up, the aphakic patients had occlusion therapy and transient rehabilitation with glasses or contact lenses. After secondary IOL implantation, they were followed up regularly for detection of uncorrected refractive errors.

## 4. Discussion

Open globe injures end in visual impairment and blindness at a significant rate in injured children and may restrict children lifelong and cause serious morbidity. The factors influencing visual outcome after ocular trauma are variable both in adults and in children. Mechanism of injury, location of injury, initial visual acuity, presence of relative afferent pupillary defect, hyphema, endophthalmitis, vitreous hemorrhage, and retinal detachment are among the factors that have been mostly investigated [11–16]. In some studies lens injury is proposed as a poor prognostic factor while others suggest that it does not affect the outcome [15]. The pattern and burden of visual impairment in children with open globe injury may vary from region to region and may be associated with the socioeconomic development level of the region. A study from Jamaica mentioned that 36% of the all pediatric ocular traumas were open globe injury [17]. The studies from Nigeria report that approximately 64.1–100% of open globe injuries present with an initial visual acuity of <6/60 [18, 19]. According to final visual acuity, they report that 79.4% of children become visually impaired. Of these 39.7 were blind. In a report from Iran, 48.9% had initial visual acuity <6/60 and 28.3% had final visual acuity <6/60 for all patterns of ocular injury [20]. However, these studies do not mention the factors resulting in visual impairment. In a recent study from Thailand, 40.8% of the children obtained a final visual acuity of >6/60. They reported that retinal detachment was significantly correlated with poor final visual outcome, consistent with the findings of Lee et al. [11]. In our study, 15% had visual impairment, and 35% were blind. We also found a significant relationship between visual impairment and injury zone, retinal detachment, and hyphema. A study from Canada involving 131 pediatric open globe injuries identified risk factors for final visual acuity <20/40 as age younger than 5 years, wound length, injury site, rupture, vitreous hemorrhage, and retinal detachment [12]. They did not find an association between hyphema and poor final visual outcome, inconsistent with our findings. Also contrary to their results, we showed no association between age and visual impairment, probably due to a smaller sample size in this study. In our study, visual impairment/blindness rates were similar in age groups. On the other hand, the objects that caused the injuries were different among the groups. Another study also identified mechanism of injury and length of time prior to surgery as risk factors [21]. A recent study from Australia analysing outcome of open and closed globe eye injuries in children reported wound length, wound site, and lens injury as parameters of poor visual outcome. They reported that 27 percent of open globe injuries had a final visual acuity <6/60 [22]. Our study was inconsistent with this study in terms of lens injury.

Presenting visual acuity is also reported to be predictive for visual outcome [23, 24]. However, it is not always possible to obtain a reliable initial visual acuity in children especially following a remarkable trauma experience. Another study found that initial vision was a less reliable predictor of final visual outcome [25]. In our study, initial visual acuity of 42.6% of children could not be obtained. All of them were under 8 years old, which could have made the assessment of visual acuity difficult. In others, the rate of initial visual acuity that could not be assessed changed from 24% to 32% [10, 13, 20–22]. Since the initial visual acuity is not available at all times in children, it may have a limited role for predicting the final visual outcome following open globe injury.

Relative pupillary afferent defect is used to calculate the ocular trauma score and has been also shown among the factors suggesting unfavourable visual outcome in children as well as in adult population in open globe injuries [26–28]. However, it is difficult to show the presence of relative pupillary defect in young children as it is difficult to cooperate during examination.

Following open globe injuries, presence of hyphema, zone 2 and zone 3 injury, and retinal detachment may end up with visual impairment and/or blindness in children. Therefore great care should be taken when the children are referred with hyphema, skleral injury, and retinal detachment and appropriate management should be initiated in an effort to minimize visual impairment.

## Figures and Tables

**Table 1:** Presenting and final visual acuity of children with open globe injuries.

		Final VA											
		>6/18			<3/60			6/18–3/60			Total		
		*N*	Row%	Column%	*N*	Row%	Column%	*N*	Row%	Column%	*N*	Row%	Column%
Initial VA	N/A	13	56	48	5	22	26	5	22	62.5	23	100	43
	>6/18	4	80	15	0	0	0	1	20	12.5	5	100	9
	<3/60	8	35	30	14	61	74	1	4	12.5	23	100	43
	6/18–3/60	2	67	7	0	0	0	1	33	12.5	3	100	5

Total		27	50	100	19	35	100	8	15	100	54	100	100

VA: visual acuity, *N*: number, %: percent, N/A: not available, Row%: the distribution of percent of patients in each initial visual acuity group according to final visual acuity groups, and Column%: the distribution of percent of patients in each final visual acuity group according to initial visual acuity groups.

**Table 2:** Subjects that cause open globe injuries.

Cause of injury	*N*	%
Metal (knife, scissors, fork, wire, and foreign body)	28	51.8
Wood (branch of tree, plank, and pencil)	8	14.8
Glass	7	13.0
Others (cable, toy, needle, and edge of a locker)	4	7.4
N/A	7	13.0
Total	54	100.0

*N*: number, %: percent, and N/A: not available.

**Table 3:** The relationship between decreased vision and initial examination findings.

		*P* value^*∗*^	Vision					
			>6/18			<6/18		
			*N*	Row%	Column%	*N*	Row%	Column%
Injury zones	Zone 1	0.034	22	62.9	81.5	13	37.1	48.1
	Zone 2		3	23.1	11.1	10	76.9	37.0
	Zone 3		2	33.3	7.4	4	66.7	14.8

Traumatic cataract	No	0.102	16	61.5	59.3	10	38.5	37.0
	Yes		11	39.3	40.7	17	60.7	63.0

Hyphema	No	0.050	24	57.1	88.9	18	42.9	66.7
	Yes		3	25.0	11.1	9	75.0	33.3

Iris injury	No	0.362	21	53.8	77.8	18	46.2	66.7
	Yes		6	40.0	22.2	9	60.0	33.3

Retinal detachment	No	0.022	26	56.5	96.3	20	43.5	74.1
	Yes		1	12.5	3.7	7	87.5	25.9

^*∗*^Pearson Chi-square test.

%: percent, N/A: not available, Row%: the distribution of percent of patients in each initial visual acuity group according to final visual acuity groups, and Column%: the distribution of percent of patients in each final visual acuity group according to initial visual acuity groups.

## Additional Points

This study is about the main causes of childhood blindness from a developing country. The authors mentioned the effects of open globe injuries on visual impairment and blindness.

## References

[1] World Health Organization (2007). *VISION 2020 Action Plan 2006–2011*.

[2] Gogate P., Kalua K., Courtright P. (2009). Blindness in childhood in developing countries: time for a reassessment?. *PLoS Medicine*.

[3] Strahlman E., Elman M., Daub E., Baker S. (1990). Causes of pediatric eye injuries. A population-based study. *Archives of Ophthalmology*.

[4] Soylu M., Demircan N., Yalaz M., Işigüzel I. (1998). Etiology of pediatric perforating eye injuries in Southern Turkey. *Ophthalmic Epidemiology*.

[5] DeRespinis P. A., Caputo A. R., Fiore P. M., Wagner R. S. (1989). A survey of severe eye injuries in children. *The American Journal of Diseases of Children*.

[6] Abbott J., Shah P. (2013). The epidemiology and etiology of pediatric ocular trauma. *Survey of Ophthalmology*.

[7] MacEwen C. J., Baines P. S., Desai P. (1999). Eye injuries in children: the current picture. *British Journal of Ophthalmology*.

[8] Moreira C. A., Debert-Ribeiro M., Belfort R. (1988). Epidemiological study of eye injuries in Brazilian children. *Archives of Ophthalmology*.

[9] Poon A. S., Ng J. S., Lam D. S., Fan D. S., Leung A. T. (1998). Epidemiology of severe childhood eye injuries that required hospitalisation. *Hong Kong Medical Journal*.

[10] Pieramici D. J., Sternberg P., Aaberg T. M. (1997). A system for classifying mechanical injuries of the eye (globe). The Ocular Trauma Classification Group. *American Journal of Ophthalmology*.

[11] Lee C.-H., Lee L., Kao L.-Y., Lin K.-K., Yang M.-L. (2009). Prognostic indicators of open globe injuries in children. *American Journal of Emergency Medicine*.

[12] Bunting H., Stephens D., Mireskandari K. (2013). Prediction of visual outcomes after open globe injury in children: a 17-year Canadian experience. *Journal of AAPOS*.

[13] Schörkhuber M. M., Wackernagel W., Riedl R., Schneider M. R., Wedrich A. (2014). Ocular trauma scores in paediatric open globe injuries. *British Journal of Ophthalmology*.

[14] Al-Mezaine H. S., Osman E. A., Kangave D., Abu El-Asrar A. M. (2010). Prognostic factors after repair of open globe injuries. *Journal of Trauma-Injury Infection & Critical Care*.

[15] Yalcin Tök O., Tok L., Eraslan E., Ozkaya D., Ornek F., Bardak Y. (2011). Prognostic factors influencing final visual acuity in open globe injuries. *The Journal of Trauma*.

[16] Teixeira S. M., Bastos R. R., Falcão M. S., Falcão-Reis F. M., Rocha-Sousa A. A. (2014). Open-globe injuries at an emergency department in Porto, Portugal: clinical features and prognostic factors. *European Journal of Ophthalmology*.

[17] Mowatt L., McDonald A., Ferron-Boothe D. (2012). Paediatric ocular trauma admissions to the University Hospital of the West Indies 2000–2005. *The West Indian Medical Journal*.

[18] Okoye O., Ubesie A., Ogbonnaya C. (2014). Pediatric ocular injuries in a resource-deficient rural mission eye hospital in Southeastern Nigeria. *Journal of Health Care for the Poor and Underserved*.

[19] Ojabo C. O., Malu K. N., Adeniyi O. S. (2015). Open globe injuries in Nigerian children: epidemiological characteristics, etiological factors, and visual outcome. *Middle East African Journal of Ophthalmology*.

[20] Aghadoost D., Fazel M. R., Aghadoost H. R. (2012). Pattern of pediatric ocular trauma in Kashan. *Archives of Trauma Research*.

[21] Baxter R. J., Hodgkins P. R., Calder I., Morrell A. J., Vardy S., Elkington A. R. (1994). Visual outcome of childhood anterior perforating eye injuries: prognostic indicators. *Eye*.

[22] Kadappu S., Silveira S., Martin F. (2013). Aetiology and outcome of open and closed globe eye injuries in children. *Clinical and Experimental Ophthalmology*.

[23] Tok O., Tok L., Ozkaya D., Eraslan E., Ornek F., Bardak Y. (2011). Epidemiological characteristics and visual outcome after open globe injuries in children. *Journal of AAPOS*.

[24] Al-Mahdi H. S., Bener A., Hashim S. P. (2011). Clinical pattern of pediatric ocular trauma in fast developing country. *International Emergency Nursing*.

[25] Rudd J. C., Jaeger E. A., Freitag S. K., Jeffers J. B. (1994). Traumatically ruptured globes in children. *Journal of Pediatric Ophthalmology and Strabismus*.

[26] Kuhn F., Maisiak R., Mann L., Mester V., Morris R., Witherspoon C. D. (2002). The ocular trauma score (OTS). *Ophthalmology Clinics of North America*.

[27] Gupta A., Rahman I., Leatherbarrow B. (2009). Open globe injuries in children: factors predictive of a poor final visual acuity. *Eye*.

[28] Meng Y., Yan H. (2015). Prognostic factors for open globe injuries and correlation of ocular trauma score in Tianjin, China. *Journal of Ophthalmology*.

